# Similar but Different: Psychological and Psychopathological Features of Primary and Secondary Hikikomori

**DOI:** 10.3389/fpsyt.2019.00558

**Published:** 2019-08-09

**Authors:** Iryna Frankova

**Affiliations:** Medical Psychology, Psychosomatic Medicine and Psychotherapy Department, Bogomolets National Medical University, Kyiv, Ukraine

**Keywords:** prolonged social withdrawal, hikikomori, primary hikikomori, secondary hikikomori, psychopathology

## Abstract

Recently, there has been an increase in reports of hikikomori around the globe, and Ukraine is not an exception. The development of hikikomori is often spurred by a history of aversive or traumatic childhood experience, for example, dysfunctions between parents or between a parent and a child (ambivalent attachment) and difficulties at school (peer rejection). Previously described models of hikikomori development mostly were based on research of mixed cohorts of patients (with and without psychiatric comorbidity). To test whether there was a difference in psychological and psychopathological features between primary hikikomori (HG1, *n* = 13) and secondary hikikomori (HG2, *n* = 22) cases comorbid with neurotic, somatoform, and stress-related disorders (F40–48, ICD-10), they were compared with each other and with a healthy control group (CG, *n* = 28). Sociodemographic data, alexithymia [Toronto Alexithymia Scale (TAS-26)], traumatic life events [life experience questionnaire (LEQ)], hostility [Buss–Durkee Hostility Inventory (BDHI)], quality of life [Chaban Quality of Life Scale (CQLS)], and personality traits (Leonhard–Schmieschek Questionnaire) were evaluated. No relevant or statistically significant differences have been found between primary and secondary hikikomori cases, except for greater hostility in the latter. When compared with the healthy control group, the primary hikikomori cases were found to have higher frequency of alexithymia, life span traumatic events (7 ± 3.6), as well as higher levels of resentment and verbal hostility, and a bigger aggression index. In secondary hikikomori cases, higher irritability and resentment have been observed, with more dysthymia, excitability, and anxiety; and although the frequency of psychological traumas was lower (5.5 ± 4), it was still significant. Primary and secondary hikikomori had largely similar characteristics in the Ukrainian sample studied, but more studies with larger samples are needed to validate generalizability of the findings.

## Introduction

Nowadays, people are increasingly connected digitally, but the prevalence of loneliness (perceived social isolation) also appears to be rising (1). One of the antecedents of social isolation is negative (traumatic) life experience, which may increase feeling of emotional loneliness, leading to loss of bonds in relationships with the close ones and self-destructive behavior (2). Recently, there have been increasingly more reports of particular form of severe and prolonged social withdrawal (i.e., hikikomori) around the globe, including Ukraine (3, 4). Since the first reference of this phenomenon in the scientific literature, there were numerous attempts to accurately translate and define the meaning of that Japanese word; therefore, various terms describe the same behavior (acute, severe, prolonged, or youth social isolation or withdrawal). Typically, hikikomori is defined as a state of social withdrawal combined with avoidance of major social interactions or responsibilities (e.g., education, employment, and friendships) lasting at least 6 months (5). Individuals with hikikomori commonly have a history of psychiatric comorbidity, but idiopathic (primary) hikikomori also exists (6).

Previous studies mostly examined the psychiatric background of individuals with hikikomori, and comorbidity with psychiatric diagnosis varies depending on study methodology and sampling. The most commonly comorbid diagnoses include schizophrenia and other psychotic disorders, as well as neurotic, mood, and anxiety disorders, such as major depression and social phobia, obsessive-compulsive disorder, eating disorders, and pervasive developmental disorders. Some researchers have mentioned autism spectrum disorder, personality disorders (such as schizoid or avoidant disorders), cannabis abuse with amotivational syndrome, or even Internet addiction (7–9). Recent international survey has shown that the most common comorbidity of hikikomori is avoidant personality disorder. Thus, it was later postulated that avoidant personality is the personality underpinning hikikomori (6, 10).

The existence of a link between hikikomori and psychiatric disorders is still under debate (11). The results of a recent 12-month study of the hikikomori (with only one case from 190 cases having no associated pathology) support the hypothesis that the phenomenon of prolonged social isolation is a severe syndrome common to different mental disorders (secondary hikikomori) and not a new diagnostic category (12).

On the other hand, a large epidemiological study aimed to clarify the correlations between hikikomori lifetime prevalence and demographics and its psychiatric background (mood, anxiety, impulse control, and substance use disorder comorbidity) has shown that of 1,660 aged from 20 to 49 years, 19 people (1.2%) had experienced hikikomori (13). With respect to diagnosis, Koyama stated that 45.5% of hikikomori cases had no lifetime experience of a psychiatric disorder, which is known as primary hikikomori.

Primary hikikomori cases often become treatment resistant: pharmacological treatment has no or partial effect on social withdrawal (14). Although a consensus diagnostic and treatment approach has not been established yet, in order to develop an optimal strategy of prolonged social withdrawal management, more research in psychopathology of primary hikikomori is needed. In contrast, previously described models of prolonged social withdrawal development were based predominantly on research of mixed cohorts of patients (15, 16). Psychological and psychopathological features of hikikomori described based on mixed cohorts are shyness; ambivalent attachment styles and life experiences including rejection by peers and parents; high loneliness and impaired social networks (deficient in social support); apparent inability to maintain meaningful social ties; social withdrawal and avoidance of real-world human interactions; and tendency toward indirect interpersonal exchanges *via* the Internet (15, 17).

Clinical characteristics of secondary hikikomori derived from a comparison of social anxiety disorder (SAD) patients with or without hikikomori were as follows: i) SAD onset preceded or coincided with hikikomori, ii) hikikomori SAD patients subset appeared to have a more severe form of SAD, and iii) hikikomori SAD patients had significantly earlier onset and had worse symptoms based on Liebowitz Social Anxiety Scale (18).

The concept of primary hikikomori is important, as one cannot understand the basis of this pathology by considering hikikomori only in relation to other disorders. Previously, the following five pathological features of primary hikikomori cases based on a comparison with patients with apathy syndrome, “taijin kyofu sho,” and personality disorders, were identified: a) episodes of defeat without a struggle, b) an ideal self-image originating in the desires of others rather than in one’s own desire, c) preserving the ideal image of the “expected” self, d) parents’ investment in the ideal self of the child, and e) avoidant behavior to maintain the positive opinion of others. Researchers highlighted the importance of family relationship problems in the onset of primary social withdrawal (9).

Overall, the development of hikikomori is often spurred by a history of aversive or traumatic childhood experience, for example, dysfunctions between parents or between a parent and a child and difficulties at school. Maladaptive attempts to deal with previous trauma might lead to neurotic, stress-related, and somatoform disorders, which are a large overall group of conditions in ICD-10 that manifest with a range of psychological and somatic symptoms (19).

If a traumatic event is a risk factor for secondary hikikomori comorbid with neurotic, stress-related, and somatoform disorders, as well as for primary hikikomori, the objective is to test whether there are any differences in psychological and psychopathological features characteristic for them.

## Materials and Methods

### Participants

The target population was inpatients or outpatients who had a history of current prolonged social withdrawal (more than 6 months). Participants (aged between 18 and 40) were recruited in 2014–2017 at the psychosomatic medicine and psychotherapy department of the Kyiv Railway Clinical Hospital No. 1 Branch of “Health Center” of JSC “Ukrainian Railway,” where patients from all other railway hospitals of Ukraine (eight branches) are referred to on a regular basis. Furthermore, to increase the range of participants, an online advertisement about checking the symptoms of hikikomori was placed in social media. The age- and sex-matched control group participants were recruited among healthy volunteers (28 persons).

### Procedure

A total of 56 patients met all the research criteria for hikikomori (17). Twenty-one patients did not complete psychological assessment or did not give consent to take part in further research, which demanded additional visit to the research center. Based on inpatient medical history and outpatient medical charts (clinical diagnosis and ICD-10 code data), the hikikomori group was divided into two subgroups: primary hikikomori, without comorbidity (HG1, *n* = 13), and secondary (HG2, *n* = 22) hikikomori, exclusively comorbid with neurotic, somatoform, and stress-related disorders (F40–F48). Primary clinical psychiatric assessment and diagnosis of mental disorders were determined in accordance with the *ICD-10 Classification of Mental and Behavioural Disorders: Clinical Descriptions and Diagnostic Guidelines* (20).

### Measures

A semi-structured interview was administered by a researcher to assess the presence of suspected hikikomori according to research hikikomori criteria (17). The sociodemographic data obtained included gender, age, education level, family status, employment (**Table 1**). Self-report questionnaires evaluated alexithymia [Toronto Alexithymia Scale (TAS-26)] (21), hostility and self-destructive behavior [Buss–Durkee Hostility Inventory (BDHI)] (22), and quality of life [Chaban Quality of Life Scale (CQLS)] (23). Other questionnaires used included Leonhard–Schmieschek Questionnaire (24), which aimed to identify accentuated personality traits, and traumatic life experience questionnaire (LEQ) (25). The latter is divided into a series of questions that relate to criminal or civil violence (robbery and torture), traffic accidents, occupational trauma, natural disasters (e.g., technical catastrophes), sexual assault or physical violence (both victim or witness), serious injury, major medical illness or threat of death (own experience or learning that trauma occurred to a close person), or adverse childhood events [separation or loss, time spent in foster care, parental divorce, significant poverty, severe mental illness, or drug addiction of a parent(s)]. LEQ is a favorable instrument as it assesses multiple types of trauma and includes a large number of potential trauma areas (38 situations in total). The parameters used to quantify the traumas include the type of trauma, the age of trauma onset, the frequency of traumatic events (the total number), the effect that the trauma had on the victim’s life during the previous year (with a range of scores from 1 to 5: no impact, mild, moderate, severe, and extreme impact) and trauma index—a sum total of the impact scores divided by the number of traumatic events.

**Table 1:** Sociodemographic characteristics of research contingent.

	CG (*n* = 28)		HG1 (*n* = 13)		HG2 (*n* = 22)	
	*N*	(%)	*N*	(%)	*N*	(%)
**Gender**						
Female	**16**	(57.2)	**7**	(53.8)	**14**	(63.6)
**Age**						
Under 21 years	**10**	(35.8)	**5**	(38.5)	**6**	(27.2)
21–26 years	**6**	(21.4)	**2**	(15.4)	**8**	(36.4)
Over 26 years	**12**	(42.8)	**6**	(46.1)	**8**	(36.4)
**Education**						
Secondary drop out	**0**	(0)	**0**	(0)	**1**	(4.5)
Secondary completed	**12**	(42.8)	**2**	(15.5)	**3**	(13.6)
Vocational training	**4**	(14.3)	**1**	(7.7)	**1**	(4.5)
University drop out	**3**	(10.7)	**5**	(38.4)	**9**	(41)
University completed	**9**	(32.2)	**5**	(38.4)	**8**	(36.4)
**Family status**						
Single	**11**	(39.3)	**11**	(84.6)	**13**	(59.2)
In relationship	**11**	(39.3)	**1**	(7.7)	**6**	(27.2)
Married	**5**	(17.7)	**1**	(7.7)	**3**	(13.6)
Divorced	**1**	(3.7)	**0**	(0)	**0**	(0)
**Employment**						
Unemployed	**10**	(35.8)	**7**	(53.8)	**11**	(50)
Part-time	**8**	(28.4)	**4**	(30.8)	**4**	(18.2)
Full-time	**10**	(35.8)	**0**	(0)	**4**	(18.2)
Freelance	**0**	(0)	**2**	(15.4)	**3**	(13.6)

### Analytic Strategy

To determine the specificity of the research subgroups, they were compared with each other and with the control group. Data analysis was performed using the Mann–Whitney test, which gives the most accurate estimates of the significance for small sample sizes and when the data do not approximate a normal distribution. Three levels of statistical significance (*p*-value) were used (*p* ≤ 0.001, *p* ≤ 0.01, and *p* ≤ 0.05). The statistical analysis was also performed to identify differences in psychological or psychopathological features of hikikomori depending on sociodemographic data. To test whether there were gender differences, the Mann–Whitney test was used. To analyze differences in age groups, age groups of social withdrawal onset, and among occupational statuses, the Kruskal–Wallis test was used, which is a preferable alternative to one-way ANOVA when the sample size is small, the variable is not normally distributed, or a standard deviation differs.

## Results

As seen in **Table 1**, 21 hikikomori cases were female, with a difference in distribution among primary and secondary subgroups. Among the participants, whose mean age was 25.6 ± 6 years, hikikomori manifested before 18 years old in 38.4% of primary and 41% of secondary hikikomori cases, and in 42.4% of all cases between 18 and 26 years old. Prolonged social withdrawal obstructed from completing academic studies (40% had not finished university); the loneliness rate (absence of relationships) constituted 80.1%; half of the hikikomori cases were unemployed; and some (14.4%) chose to work as freelancers. There were statistically significant differences depending on sociodemographic data. The level of suspicion [χ^2^(2) = 5.98, *p* = 0.05] and dysthymia [χ^2^(2) = 5.9, *p* = 0.05] was significantly higher in those hikikomori cases who were younger than 26 years old. The level of anxiety was significantly lower in those who worked freelance [χ^2^(2) = 9.93, *p* = 0.02]. Depending on the age of hikikomori onset, the earlier social withdrawal occurred the higher the level of trauma impact was [χ^2^(2) = 5.4, *p* = 0.06]. Women had higher levels of emotional lability (*p* ≤ 0.01), anxiety and experienced a larger impact of the life span traumatic events (*p* ≤ 0.05).

The secondary hikikomori subgroup (HG2) had the following comorbid psychiatric disorders: adjustment disorder (22.7%), generalized anxiety disorder (18.2%), post-traumatic stress disorder (13.7%), social phobia (13.7%), obsessive-compulsive disorder (13.7%), panic disorder (9%), somatoform disorder (9%), and dissociative identity disorder (4.5%).

When comparing HG1 with HG2, only one statistically significant difference was found in physical hostility (BDHI) 5.9 ± 2 vs 4.3 ± 2.3, but the comparison of HG1 and HG2 with CG showed different statistically significant findings (**Table 2**). Compared with CG, HG1 had higher levels of alexithymia (61.5% vs 42.9% HG2, 17.2% CG), and according to LEQ, they reported higher numbers of life span traumatic events and their greater impact.

**Table 2:** Statistically significant differences between HG1, HG2, and CG (Mann–Whitney *U* test).

Scale	Characteristics	HG1	HG2	CG	CG–HG1		CG–HG2		HG1–HG2	
		*M* ± *SD*	*M* ± *SD*	*M* ± *SD*	*U*	p*-value	*U*	p*-value	*U*	p*-value
TAS-26	Alexithymia	73.4 ± 9	70.8 ± 11	61.2 ± 13	78.5	0.002	194.5	0.02	112	0.2
BDHI	Irritability	6.9 ± 2.5	7.1 ± 2.1	6 ± 1.8	133.5	0.1	188	0.01	137	0.8
	Resentment	5.5 ± 1.5	5.1 ± 1.8	3.8 ± 2	98.5	0.01	206	0.04	127.5	0.6
	Verbal hostility	7.8 ± 2.9	6.6 ± 2.7	5.8 ± 2.7	107.5	0.03	242.5	0.1	109.5	0.2
Leonhard–Schmieschek Questionnaire	Physical hostility	5.9 ± 2	4.3 ± 2.3	4.8 ± 2.6	137	0.2	296	0.4	82	0.03
	Negativism	2.9 ± 1.2	2.1 ± 1.5	3.1 ± 2.5	178	0.9	224.5	0.09	98	0.1
	Excitability	14.2 ± 5.4	15.4 ± 5.7	12.1 ± 4.6	146	0.3	182	0.02	112.5	0.3
	Dysthymia	12.7 ± 5.3	14.8 ± 3.9	9.6 ± 4.6	130	0.1	135	0.001	109	0.2
	Anxiety	14.3 ± 5	14.7 ± 6.3	10.6 ± 5.9	116	0.06	192	0.02	130.5	0.6
LEQ	Number of traumatic events	7 ± 3.6	5.48 ± 4	4.4 ± 3.5	87.5	0.006	227	0.1	103	0.1
	Impact of traumatic events	22 ± 14	16 ± 13.6	11 ± 10.6	84	0.005	198	0.03	104.5	0.1
	Trauma index	2.98 ± 0.8	2.97 ± 1	2.23 ± 1	96	0.01	174	0.009	139	0.8
CQLS	Quality of life	13.7 ± 3.3	11.7 ± 2.7	19.3 ± 3.5	45.5	0.001	22.5	0.001	114	0.3

*Exact Sig. [2 * (1-tailed Sig).]—the exact significance level p-value but not corrected for ties.

**Figure 1:**
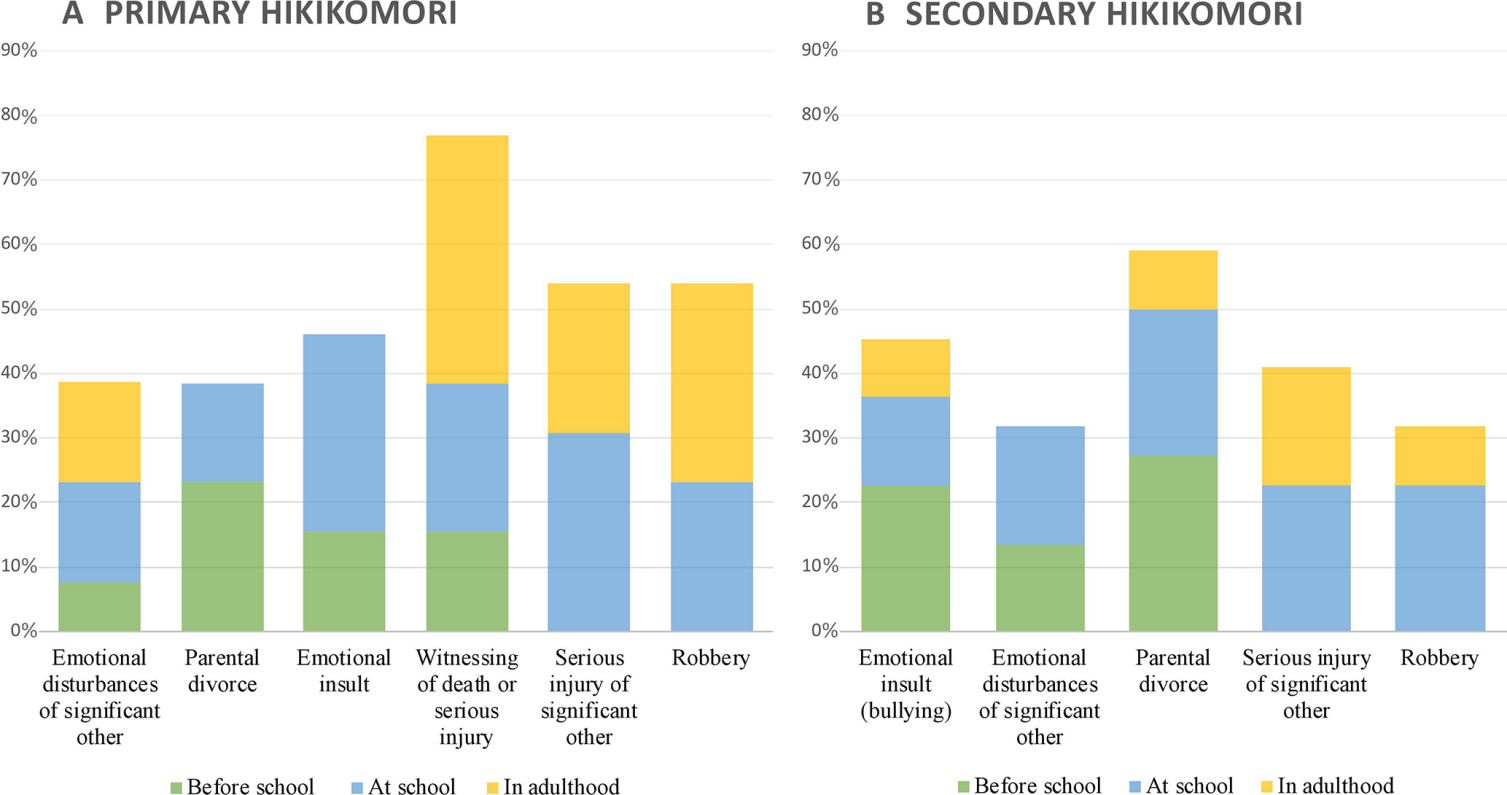
Prevalence (%) and impact of traumatic and life adverse events in primary and secondary hikikomori. Most influential past experiences are illustrated on **A** and **B** and are arranged so as to reflect their actual impact, with the most influential experiences on the left.

The trauma indices in both research groups, as well as the distribution of post-traumatic stress (61.2% vs 61.9%), were almost equal. The most prevalent resembling traumatic situations for hikikomori included emotional insults or neglect (54.3%), learning about a serious life-threatening injury or an unexpected death of a close person (45.7%), emotional disturbances of significant others (42.9%), or parental divorce (34.3%). HG1 faced psychological traumas 25% and 60% more frequently than do HG2 and CG, respectively; in contrast, secondary hikikomori cases witnessed serious injury or death 4.5 times more frequently (**Figure 1**).

The majority of primary (70%) and nearly half of secondary hikikomori cases had higher than normal index of hostility (*M* = 12.08 ± 6.6 vs *M* = 10.27 ± 3.1), and the comparison of HG1 and CG has shown that primary hikikomori cases had higher levels of resentment and verbal hostility, and a bigger aggression index. At the same time, secondary hikikomori cases had higher irritability and resentment than had healthy controls. There was no statistically significant difference between HG1 and CG in accentuated personality traits, but HG2 had significantly higher levels of dysthymia, excitability, and anxiety. According to CQLS, nearly half of HG1 (48.6%) and HG2 (46.2%) evaluated their quality of life as low.

## Discussion

To our knowledge, this study is the first to analyze a sample of hikikomori cases in Eastern Europe, thereby providing the justification for the hypothesis that this condition arises from specific socioeconomic and cultural changes in the modern society, not just a Japanese culture-bound social withdrawal syndrome (26). Irrespective of the etiology of withdrawal, whether it stems from other primary mental health problems or is an idiopathic case, modern Internet-connected world allows patients to commit “social suicide,” as the Internet can satisfy all the needs of those who want to remain alone in their rooms isolated in their “virtual tombs.” Research has shown that the level of anxiety might depend on occupational status, and it was lowest in those who work as freelancers, which seems to be a specific coping strategy for people trying to avoid social interactions and responsibilities.

Although a consensus diagnostic approach has not been agreed upon yet, some diagnostic criteria for hikikomori have already been revised. The age specifier (individuals aged less than 18 years) was eliminated, as research has shown that the onset of social withdrawal can occur at any age (27, 28). In the analyzed sample, in 37% of participants, the onset of social isolation occurred during adolescence, and in 40% of cases, it started between 18 and 26 years old.

Another important finding was that the levels of suspicion and dysthymia were higher in those hikikomori cases who were younger than 26 years. This period in life is characterized by movement, changes, and transitions from one state into another in several domains at the same time. Young people have to make decisions about important concrete directions in life, for example, school, living situation, and peer group. They must also address new challenges with regard to building their own identity, developing self-esteem, acquiring increasing independence and responsibility, and building new intimate relationships. Besides, that they are often confronted with high expectations from significant relatives and peers. Such situations inevitably provoke a certain degree of helplessness, insecurity, stress, and a sense of losing control (29). To address these challenges and successfully cope with these emotions, young people must have access to significant supporting resources such as a stable living situation, intimate friendships, and sufficient economic resources (30).

In reality, however, in both primary and secondary hikikomori cases, there was a high frequency of past traumatic and life adverse events, which prevented them from overcoming the challenges described above. The majority had symptoms of post-traumatic stress (i.e., vulnerable to stress), with their experience of traumatic events being very intense and having a strong impact on their current life. However, the most prevalent situations in terms of frequency were not the most influential. As shown in **Figure 1**, experiences that affected both primary and secondary hikikomori cases the most were as follows: emotional disturbances that occurred to significant others (e.g., severe depression and chronic alcohol or drugs addiction), parental divorce, and emotional insult or neglect (humiliation, embarrassing experiences, or feeling worthless). These traumas occurred predominantly in early childhood or at school. Typically, because of a shift from perceived safety to a new environment (from school to university, moving from one city or country to another), the youth were becoming “a stranger,” “a black sheep,” or “a scapegoat” in a peer group and were experiencing bullying. These findings correspond with the psychosocial developmental theory of hikikomori (15, 31). In a situation of loss of their secure environment combined with a history of early-life adverse events (loss or separation), an individual’s social interactions are blocked due to the reactivation of the insecure attachment system and its emotional and behavioral patterns and coping strategies.

Both primary and secondary hikikomori cases might be characterized as having insufficient ability to identify and verbalize their emotions and a tendency to self-aggressive behavior especially due to high levels of resentment, inner tension, high anxiety, self-doubt, and a low quality of life. Primary hikikomori cases also resort to direct (verbal and physical) hostility, most probably as a protective behavioral reaction to their immediate environment previously described as “defeat without a struggle” (9). Hostility toward parents is commonly mentioned in literature (32); one study reported 43% of the subjects wanting to kill their parents, with 23% having physically attacked them while attempting to initiate a conversation (33).

When focusing on the differences between primary and secondary hikikomori cases, it was found that in the latter, aggression manifests itself as a personality trait (excitability and impulsivity) rather than behavior. Because of dysthymia and high anxiety, secondary hikikomori cases have a pessimistic self-perception, often become victims of bullying, prefer staying at home, assume a dependent passive position, and have fear of social communication.

It is worth mentioning that in this study the number of female subjects with secondary hikikomori was higher than the numbers found in previous reports, which suggested that social isolation was more frequent in men (13, 28, 31), whereas comorbid psychopathology in the study were neurotic, somatoform, and stress-related disorders, such as panic disorder, specific phobia, social anxiety disorder, generalized anxiety disorder, and post-traumatic stress disorder, which are known to be more prevalent among women than men (34, 35).

### Limitations

The present study has several limitations. First, the sample size was relatively small, and cases are not statistically representative of the broader Ukrainian hikikomori population. However, despite the small sample size, statistical methods were chosen accordingly. Second, in this study, secondary hikikomori group included only neurotic, somatoform, and stress-related disorders as comorbidity. Future research should try to explore differences of primary cases with another psychopathology.

## Conclusion

The findings of this research seem to validate the hypothesis that primary and secondary hikikomori had largely similar characteristics in the Ukrainian hospital sample studied. Future studies encompassing larger samples are needed to validate the generalizability of the findings.

## Ethics Statement

All subjects gave written informed consent in accordance with the Declaration of Helsinki. The protocol was approved by the Ethics Committee of “Health Center” Branch of JSC “Ukrainian Railway” Kyiv Railway Clinical Hospital No. 1, and this study was carried out in accordance with its recommendations. The study procedures were carried out in accordance with the ethical standards. No bio-markers or tissue were collected. Participation was entirely voluntary, confidential, and anonymous. Participants were informed that they were free to withdraw from the study at any time.

## Author Contributions

The author confirms being the sole contributor of this work and has approved it for publication.

## Conflict of Interest Statement

The author declares that the research was conducted in the absence of any commercial or financial relationships that could be construed as a potential conflict of interest.

## Abbreviations

TAS-26, Toronto Alexithymia Scale; LEQ, Life experience questionnaire; BDHI, Buss–Durkee Hostility Inventory; CQLS, Chaban Quality of Life Scale; HG1, primary hikikomori group; HG2, secondary hikikomori group; CG, control group.
